# DNA methylation state of the galectin-3 gene represents a potential new marker of thyroid malignancy

**DOI:** 10.3892/ol.2013.1312

**Published:** 2013-04-18

**Authors:** SIMONA KELLER, TIZIANA ANGRISANO, ERMANNO FLORIO, RAFFAELA PERO, MIRIAM DECAUSSIN-PETRUCCI, GIANCARLO TRONCONE, MARIO CAPASSO, FRANCESCA LEMBO, ALFREDO FUSCO, LORENZO CHIARIOTTI

**Affiliations:** 1 Department of Cellular and Molecular Biology and Pathology, University of Naples, Institute of Experimental Endocrinology and Oncology CNR, Naples, Italy;; 2 Department of Pathology, University of Lyon 1, Pierre Bénite, France;; 3 Center of Advanced Biotechnology CEINGE, University of Naples, Naples, Italy; 4 Department of Pharmaucetical and Toxicological Chemistry, Faculty of Pharmacy, University of Naples, Naples, Italy

**Keywords:** galectin-3, human thyroid cancer, DNA methylation, tumor marker

## Abstract

In order to supplement the cytopathological assessment of thyroid tumors, there is a need for new markers to correctly diagnose malignant thyroid lesions and avoid unnecessary and potentially harmful therapies for patients. The immunohistochemical expression of galectin-3 is currently considered to be the most accurate stand-alone marker for thyroid cancer diagnosis. The aim of this study was to establish whether the methylation state of the galectin-3 gene is a candidate molecular marker for thyroid malignancy. Thyroid specimens from 50 patients were analyzed, including 5 normal thyroid, 3 goiters, 39 papillary and 3 anaplastic thyroid carcinoma cases. High-resolution methylation analyses was performed to investigate the methylation state of a large genomic region (from −89 to +408) encompassing the galectin-3 transcriptional start site. Within this region, 5 CpG sites (nucleotide positions +134, +137, +142, +147 and +156) were observed to be differentially methylated among the samples and were further analyzed by the quantitative pyrosequencing technique. The hypomethylation of the +134, +137, +142, +147 and +156 CpG sites was observed to be markedly associated with cancer. Although the methylation degree of each single site was highly variable in non-neoplastic tissues, the average methylation state of the 5 CpG sites clearly distinguished cancer from the nonneoplastic thyroid tissues.

## Introduction

Thyroid cancer is the most frequently occurring endocrine malignancy. Since this cancer often afflicts young adults, thyroid cancer represents a challenging clinical problem. Thyroid carcinomas are derived from follicular epithelial cells and have a broad spectrum of neoplastic phenotypes. These phenotypes are well-differentiated thyroid carcinoma, including papillary thyroid carcinoma (PTC) and follicular thyroid carcinoma (FTC), poorly differentiated thyroid carcinoma, representing ∼5% of thyroid cancers, and the rare but always rapidly lethal anaplastic thyroid carcinoma (ATC) (1). Although thyroid neoplasms may be diagnosed from fine needle aspirates, differentiating the more frequently occurring follicular adenoma from malignant lesions remains challenging. There are currently no markers to separate these groups; therefore the majority of patients are subjected to surgery and radiotherapy. PTC is associated with mutations in RET, BRAF and RAS, while FTC exhibits either RAS mutations or PPARg gene rearrangements (2–6). The search for genetic alterations for the identification of malignancy has low sensitivity, since numerous cancer samples do not bear any of these genetic alterations, and low specificity, since benign adenoma shares genetic lesions (RAS, PPARg and RET/PTC) with cancer (7). Immunohistochemical studies of thyroid cancer have allowed the development of potential molecular diagnostic tools (8). Galectin-3 (Gal-3) has received significant attention and is considered to be the most accurate stand-alone marker for differentiated thyroid cancer diagnosis. Gal-3 is highly expressed in thyroid cancer, but not in normal thyroid tissue and infrequently in benign thyroid lesions (9). In a large scale study, Gal-3 was reported to have a sensitivity of 83%, specificity of 92% and accuracy of 95% (10). Despite initial enthusiasm, extensive experience with Gal-3 as a potential marker of malignancy by immunocytochemistry has failed to provide clear evidence of superior diagnostic accuracy compared with traditional cytology (11). Clearly, there is a need for additional markers in order to accurately diagnose malignant thyroid lesions and avoid patients undergoing unnecessary and potentially harmful therapies.

The DNA methylation state of several tumor suppressor genes has been proposed to be an advantageous marker of malignancy in various tumor types (12,13). Aberrant hypermethylation and the consequent silencing of tumor suppressor genes have been frequently observed in thyroid cancer (14–16) but failed as a selective molecular marker in thyroid tumorigenesis. However, at present the possibility that genes that are silent in the normal thyroid but specifically activated in thyroid malignancy are subject to cancer-specific epigenetic alterations, has not yet been investigated.

The present study addressed the hypothesis that the DNA methylation state of Gal-3 gene may be associated with malignancy in thyroid neoplasias.

## Materials and methods

### Tissue samples

Neoplastic and normal human thyroid tissues were obtained from surgical specimens and immediately frozen in liquid nitrogen. Thyroid samples were collected at the Service d’Anatomo-Pathologie (Centre Hospitalier Lyon Sud, Pierre Benite, France). The study was approved by the ethics committee of the University of Naples, Naples, Italy.

### DNA extraction from tissues

Genomic DNA was extracted for each sample from a portion of liquid nitrogen-pulverized tissue and was prepared using a QIAamp DNAMini kit (Qiagen, Hilden, Germany), following the manufacturer’s instructions.

### Bisulfite treatment

The sodium bisulfite conversion was performed using an EZ DNA Methylation kit (Zymo Research, Irvine, CA, USA). The manufacturer’s instructions were followed by using 2 mg of genomic DNA and eluting in 30 ml of H_2_O.

### MassARRAY methylation analysis

MassCLEAVE biochemistry was performed as described previously (17,18). Mass spectra were acquired using a MassARRAY Compact matrix-assisted laser desorption/ionization-time-of-flight (MALDI-TOF) mass spectrometer (Sequenom, San Diego, CA, USA) and spectra methylation ratios were generated by the Epityper software version 1.0 (Sequenom).

The primers used in the present analysis were: Gal3m forward, 5′-aggaagagagTTTATTTAGGTGATTTTG GAGAGGG-3′; and Gal3m reverse, 5′-cagtaatacgact cactatagggagaaggctAAAAACAAAACACAAACTATAAAA CTCTC-3′. For reverse primer, an additional T7 promoter tag for *in vivo* transcription was added, as well as a 10-mer tag on the forward primer to adjust for melting temperature differences. Sequences of these tags are indicated in lower case. The presence of CpG islands in the genomic region analyzed was assessed using the CGplot software (http://www.ebi.ac.uk/emboss/cpgplot/).

### Pyrosequencing methylation analysis

Quantitative DNA methylation analysis was performed using a Pyrosequencing PSQ 96MA (Biotage AB, Uppsala, Sweden) following the manufacturer’s instructions. The reactions were assayed on the PSQ 96MA using the provided SNP analysis software. The primers used for the PCR reactions were: LGalS3 forward, 5′-GGTTCGGGGAGAGGATTGGT-3′; and LGalS3 reverse, 5′-ATAACTCCAAACCTCAAATACTCC-3′ (5′-biotinylated). Amplifications were performed using the protocols developed previously (19,20). The sequencing primer (LGalS3S1) was 5′-AGGATTGGTTGGGTAG-3′. The target CpGs were evaluated by anlalyzing the resulting pyrograms. Analysis of a non-CpG cytosine was used as an internal control for the completeness of the bisulphite treatment.

### Statistical analysis

The statistical significance of differences between the groups was assessed by the Student’s t-test. Data were expressed as the mean ± standard deviation (SD). The variance on the variable among the groups was calculated by Levene’s test for the equality of variance. All experiments were repeated at least three times. The methylation score was the sum of the methylation value for each CpG site (nucleotide positions +134, +137, +142, +147 and +156) of each patient. P<0.05 was considered to indicate statistically significant differences.

### Databases

The Gal-3 (LGALS3) gene sequences were retrieved by the Ensembl database accession number, ENST0000025430.

## Results

### Gal-3 gene methylation analysis

**Figure 1.**
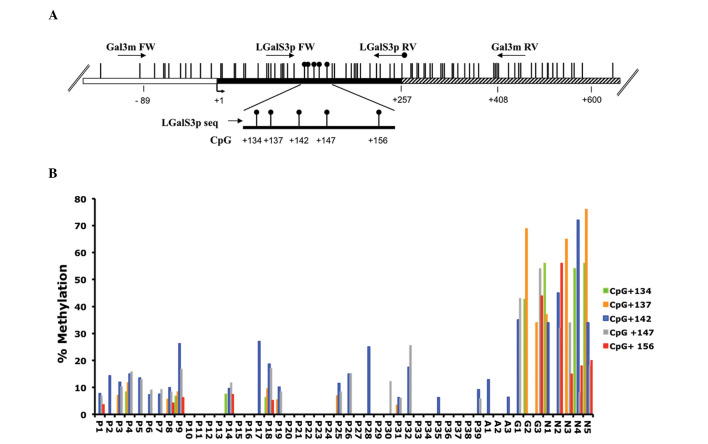
Gal-3 gene methylation analysis. (A) Structure of the human Gal-3 promoter gene. The transcriptional start site (+1) is indicated by an arrow. The regulatory upstream region (white box), exons (black) and first intron (striped box) are indicated. Vertical bars represent the relative positions of each CpG site. The primer positions used for MALDI-TOF and pyrosequencing analysis are indicated by arrows (Gal3m FW/Gal3m RV and LGalS3p FW/LGalSp RV biotinylated). Black circles represent the CpG sites analyzed by pyrosequencing (CpG +134, +137, +142, +147 and +156). (B) Histogram representing the percentage of methylation of each CpG analyzed in each sample. P1-P39, papillary thyroid carcinoma; A1-A3, anaplastic thyroid carcinoma; G1-G3, thyroid goiter; N1-N5, normal thyroid; Gal-3, galectin-3; MALDI-TOF, matrix-assisted laser desorption/ionization-time-of-flight.

DNA methylation analysis of the Gal-3 promoter region was performed on genomic DNA extracted from human tissue samples derived from 42 tumors (39 papillary and 3 anaplastic carcinomas), 3 goiters and 5 normal thyroid tissues obtained from surgical specimens. The map of the human Gal-3 gene, including the relative positions of the analyzed CpG sites, is shown in Fig. 1A. To investigate the methylation state of a Gal-3 genomic region encompassing the transcriptional start site, two independent quantitative DNA methylation analysis techniques were performed. Mass spectrometry-based methylation analysis (MassARRAY) and pyrosequencing technology were used to assess the precise degree of methylation of each CpG site. First, a large genomic region from −89 to +408, which included the transcriptional start site and 65 CpG sites, was analyzed by MassARRAY. The results of duplicate experiments indicated that, in this genomic region, the majority of the CpG sites were unmethylated or slightly methylated (data not shown) in the neoplastic and non-neoplastic tissues, with the exception of a small region, including 5 CpG sites, localized downstream of the transcriptional start site, where differential methylation was detected. However, since MALDI-TOF analysis did not allow the determination of the methylation degree of each single CpG site in this region, focused DNA methylation analysis using a different technology was performed. Pyrosequencing analysis was then performed with the aim of quantitatively evaluating the methylation state of each of the 5 CpG sites (nucleotide positions +134, +137, +142, +147 and +156). The pyrosequencing assays were performed using the primers indicated in Fig. 1A, covering the region from +104 to +196, and the results were plotted on a histogram showing the methylation degree of each CpG site in each tissue sample (Fig. 1B). Marked differential methylation was observed between the neoplastic and non-neoplastic groups. A low methylation degree (0–28%) at the 5 CpG sites was observed in all the neoplastic samples, including anaplastic and papillary carcinomas, while a high methylation degree (up to 80%) was present at a minimum of 2 out of the 5 analyzed CpG sites in the non-neoplastic tissues. Notably, the identity of the hypermethylated CpG sites in the non-neoplastic tissues was variable among the samples. This suggested that the methylation state of the whole region (+134/+156), rather than the methylation state of single CpG site, is associated with the sample groups. Thus, statistical analysis of the data was performed.

### Statistical analysis

**Figure 2.**
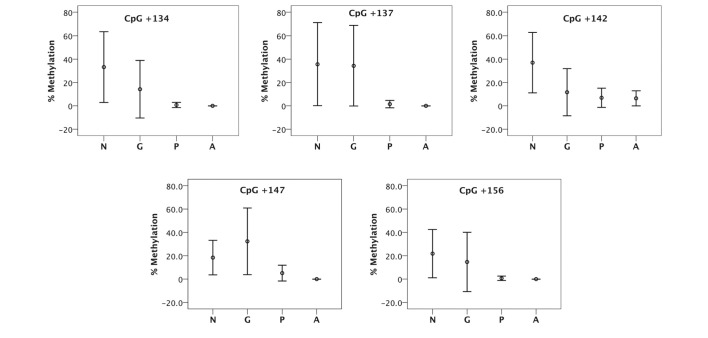
Statistical analysis of individual CpG site methylation degree. The plots represent the mean ± standard deviation of the percentage of methylation at each CpG site in each tissue. Statistical analysis was performed to verify whether the percentage of methylation was statistically significantly higher in normal thyroid and goiter compared with papillary and anaplastic thyroid carcinoma. Under the assumption of unequal variance on the variable among the groups (Levene’s test for equality of variance; P<0.003), statistically significant results were observed (Student’s t test, P= 0.02–0.06). N, normal thyroid; G, goiter; P, papillary; A, anaplastic thyroid carcinoma; Gal-3, galectin-3.

The associations between the methylation degree of each of the 5 CpG sites and tumor types were analyzed. In the first analysis, each methylated region was compared among the various thyroid tissues. Although the difference in the percentage of methylation was higher in the normal thyroid and goiter than the papillary and anaplastic thyroid carcinoma, the high variability of the normal thyroid and goiter tissues makes these results poor in terms of statistical significance and reproducibility (Fig. 2).

**Figure 3.**
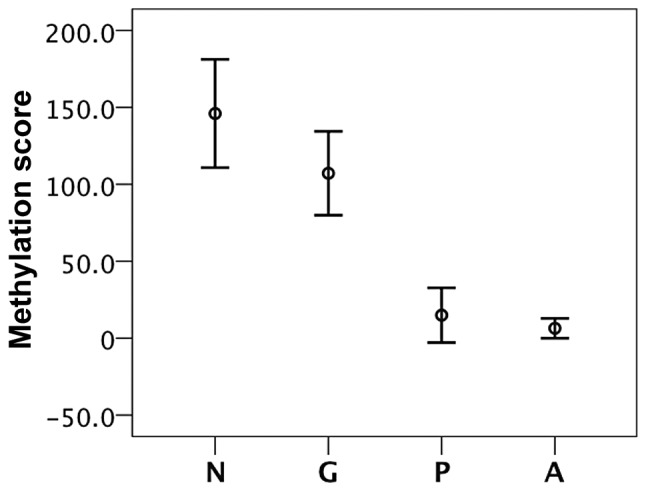
Statistical analysis of the average methylation status of 5 CpG sites. Plot represents the mean ± standard deviation of the methylation score obtained by adding the values of the CpG sites (+134, +137, +142, +147 and +156) for each patient. The statistical significance of differences between the groups (papillary vs. goiter; papillary vs. normal / anaplastic vs. goiter; and anaplastic vs. normal) was assessed by the Student’s t test. P<0.004 in all cases. The two compared groups had approximately equal variance on the variable (Levene’s test for equality of variance; P>0.05).

To obtain robust and reproducible results, a new variable named ‘methylation score’ was then created by the addition of the methylation values of the CpG sites lying in the differentially methylated region (+134, +137, +142, +147 and +156) for each patient. Statistical analysis was performed by considering the average methylation score of the CpG sites. The results, shown in Fig. 3, indicate that the average DNA methylation degree at the 5 CpG sites was significantly lower (P<0.004) in the anaplastic and papillary thyroid carcinomas compared with either the goiters or normal thyroid tissues. Moreover, a reduction of data variability was observed. The use of the methylation score, which includes more CpG methylated sites, may be a reliable diagnostic tool for distinguishing the cancer tissue from normal tissue.

## Discussion

The present study showed that the average methylation degree of 5 CpG sites in the Gal-3 gene regulatory region is significantly decreased in thyroid cancer tissues compared with non-neoplastic thyroid tissues. Although Gal-3 gene expression is an established marker of thyroid malignancy, to the authors’ knowledge this is the first report investigating the Gal-3 DNA methylation state in thyroid tumors. Previous studies have reported that aberrant hypermethylation at various genes is associated with thyroid malignancy, including genes involved in the control of cell proliferation and invasion, such as p16INK4A (21), Rassf1A (22), PTEN (23), Rap1GAP (24), TIMP3, RAR-b2, DAPK (15,16,25), CDH1 (26,27), TGFb and CITED1 (28), as well as genes specific to thyroid differentiation, such as Na^+^/I^−^ symporter (NIS), TSH receptor, pendrin, SL5A8 (29,30) and TTF-1 (31), as reviewed by Catalano *et al* (32). In the majority of these studies, which were performed by methylation-specific PCR, a considerable overlap was observed in the methylation levels between benign and malignant tumors, with the exception of hypermethylation at RAR-b2 (15,16), NIS (33), TSHR (34), ECAD (26) and ATM (35), which was observed to be more prevalent in patients with papillary thyroid carcinoma than in non-malignant thyroid diseases. However, none of the observed tumor-related gene hypermethylation is considered to be a stand-alone marker for distinguishing malignant from benign tumors. Galusca *et al* (36) evaluated the global DNA methylation status in several types of thyroid tumor using a monoclonal anti-5-methylcytidine (5-mC) antibody in an immunohisto-chemical quantitative analysis. The authors observed global DNA hypomethylation in thyroid carcinomas compared with benign lesions with an overall accuracy estimated to be similar to Gal-3 immunostaining. Notably, the combination of 5-mC and Gal-3 led to an accuracy of 96% (36). The presently reported data, obtained by high resolution methylation analysis at the gene-specific level, shows that hypomethylation of the Gal-3 gene clearly distinguishes papillary and anaplastic carcinoma from non-neoplastic thyroid tissues. Marked differential methylation was observed between the neoplastic and non-neoplastic groups. This difference was easily detectable by considering the average methylation state of the 5 CpG sites included in the Gal-3 gene region from +134 to +156, rather than considering the methylation state of individual sites. In fact, the identity of the hyper-methylated CpG sites in non-neoplastic tissues was highly variable among the samples. Although in the present study the Gal-3 methylation state was not investigated in thyroid adenomas, the data suggest that the evaluation of the Gal-3 methylation state at the five identified CpG sites may greatly aid in thyroid tumor diagnosis. Further studies on a larger range of samples, including malignant and benign thyroid tumors, are likely to clarify whether the assessment of the methylation state of CpG sites +134, +137, +142, +147 and +156, possibly in combination with Gal-3 immunostaining, may be a candidate analysis which substantially contributes to increasing the accuracy of the currently used markers for distinguishing thyroid cancer from benign thyroid adenomas.
